# Leprosy in Post-elimination Era: A Study Conducted in Rural Tertiary Settings in North India

**DOI:** 10.7759/cureus.59464

**Published:** 2024-05-01

**Authors:** Sweta S Kumar, Nisha Yadav, Anurag Yadav, Monika Srivastava, Santosh Kumar, Anuj Jain

**Affiliations:** 1 Dermatology, Uttar Pradesh University of Medical Sciences (UPUMS), Etawah, IND; 2 Anatomy, Uttar Pradesh University of Medical Sciences (UPUMS), Etawah, IND; 3 Physiology, Mahaveer Institute of Medical Sciences and Research, Bhopal, IND; 4 Respiratory Medicine, Autonomous State Medical College, Etah, IND

**Keywords:** stigma, lepra reactions, nlep, deformity, nerves, leprosy

## Abstract

Background

Leprosy is an age-old disease caused by *Mycobacterium leprae*. The disease was declared eradicated in India in 2005. Many new cases are still being identified in the outdoor patient department. This study was undertaken to understand the epidemiological, clinical, and social aspects of leprosy among new patients, and assess the current situation regarding caseload and presentation.

Material and methods

This study was designed as an observational study. It was carried out in people newly diagnosed with leprosy attending the outpatient department of Dermatology, Venereology, and Leprology in the tertiary care hospital in Uttar Pradesh University of Medical Sciences from July 2022 to January 2024. A total of 231 people afflicted with leprosy were included in the study. The data collected was statistically analyzed to identify demographic and social patterns, clinical presentations, and features associated with leprosy.

Result

Out of these 231 patients, 139 (60.17%) were male and 92 (39.83%) were female. Most cases belonged to the age group 40-59 years 87 (37.66%). History of close contact with an afflicted person was present in 34 (14.71%). Clinically, most patients belong to the borderline tuberculoid (BT) type. Only 24 (10.4%) patients were found positive for *M. leprae* by slit-skin smear examination. The ulnar nerve was the most common nerve involved in 63 (27.27%) cases. Trophic ulcers were the predominant deformity in 34 (14.7%), followed by foot drop in 13 (5.62 %).

Conclusion

The present study provides an overview of the prevailing trends of Leprosy within a specific region in the post-elimination era. The findings underscore the significance of the ongoing National Leprosy Eradication Program (NLEP) program and stress the importance of aligning them with the common goal of eliminating the burden and stigma of Leprosy from society.

## Introduction

Leprosy, also known as Hansen's disease, is a chronic but curable infectious disease caused by *Mycobacterium leprae* that is still endemic in more than 120 countries worldwide [1]. The disease usually presents as patchy lesions on the skin. It also affects peripheral nerves, eyes, and the mucosa of the upper respiratory tract [1]. The physical symptoms include flat and discolored patches of pale and reddish skin, maybe numbness in the limbs (hands and feet), and loss of sensation in the affected patch of skin, accompanied by loss of eyebrows and eyelashes in some cases [2,3].

The Ridley-Jopling classification of leprosy classifies the disease into five groups: tuberculoid (TT), borderline tuberculoid (BT), mid-borderline (BB), borderline lepromatous (BL), and lepromatous (LL) [4]. A specific type of leprosy occurs in India, people experience symptoms related to peripheral nerve involvement but no skin lesions, making diagnosis difficult. A nerve biopsy is necessary for confirmation.

WHO has classified leprosy into paucibacillary and multibacillary disease for treatment purposes. Leprosy is considered multibacillary when the affected individual has more than five skin lesions with or without nerve involvement or a skin smear is positive for *M. leprae* at any site. Paucibacillary leprosy is diagnosed if less than five skin lesions are seen with no nerve involvement and negative skin smears at all the sites [5].

When left untreated, leprosy can result in progressive and permanent damage to the skin, nerves, limbs, and eyes. However, it is curable, and treatment at the initial stages can prevent disability. In addition to physical deformities, individuals affected by leprosy also endure stigma and discrimination. WHO classification is useful from a therapeutic perspective. According to this classification, paucibacillary cases are treated for six months, while multibacillary cases are treated for 12 months, both using different treatment regimens. This difference affects measures of leprosy epidemiology [5].

Despite being declared eliminated (less than one case per 10000 people) as a global public health problem by the World Health Organization in 2000 and 2005 in India. India has the highest burden of disease (58% of new cases) in the world [6]. The national leprosy statistics are calculated in India based on the number and details of self-reported patients registered with health facilities and hospitals affiliated with the National Leprosy Eradication Program (NLEP), and the numbers detected through block and district-level active leprosy case detection campaigns of NLEP in specified geographic areas in that year.

According to official data obtained from 139 countries in the six WHO regions, 127558 new leprosy cases were detected worldwide in 2020. Of these, 8629 patients were children under 15 years of age. This data suggests that the new case detection rate stands at 4.4 per million children under 15 years of age. Out of these new patients, 7198 cases were detected with grade-2 disabilities (G2D) and the new G2D rate was recorded at 0.9 per million of the population. At the end of the year 2020, the prevalence was recorded to be 129389 active cases on treatment worldwide. The prevalence rate equates to be at 16.7 per million of the population.

The COVID-19 pandemic in 2020 effectively disrupted the ongoing program implementation worldwide, leading to a 37% reduction in new case detection compared to 2019, but this does not mean a reduction in new cases. Although the COVID-19 pandemic disrupted health services, it also provided a window to strengthen digital health initiatives for diagnosis, referral, monitoring, and staff training in several countries.

The long-term vision of WHO is to have zero leprosy, that is, zero infection and disease, zero disability, and zero stigma and discrimination. Towards this goal, NTD Roadmap 2030 and Global Leprosy Strategy 2021-2030 were envisioned, taking the Annual Leprosy Data of 2019 as a baseline for monitoring the progress. This strategy towards zero leprosy by 2030 has four pillars, namely, implementing zero leprosy road maps, scaling up of prevention of the spread of disease, managing existing cases and preventing disabilities, and combating the stigma related to the disease, with disease elimination and interruption of transmission being at the core of this strategy.

In 2020, the WHO brought out Leprosy/Hansen Disease Management of Reactions/Prevention of Disabilities, a technical guide 2020, to provide hands-on guidance to health workers. This document aims to critically assess current practices in managing leprosy reactions and neuritis, proposing concrete improvements to empower national programs in achieving their objectives of early leprosy diagnosis, effective lepra reaction management, and minimizing disability.

WHO also brought out a technical guide on leprosy (Hansen disease) contact tracing and post-exposure prophylaxis in 2020. The primary aim of this technical guide is to provide guidance to countries and programs regarding the implementation of contact screening and chemoprophylaxis using single-dose rifampicin. Additionally, the World Health Organization has developed e-learning modules designed to augment the knowledge and capabilities of staff at all levels. These modules cover a wide range of topics, including suspected referrals and diagnosis, treatment of Leprosy, and the management of associated disabilities. In India, the “Sparsh leprosy awareness campaign” was launched on 30th January 2017 to help reduce stigma and discrimination against persons suffering from leprosy [7]. Keeping this background in sight, this study was undertaken to understand the changing clinical and demographic profile of people afflicted with leprosy and analyze the burden of disease upon society.

## Materials and methods

Study design and settings

This observational study was conducted in clinically diagnosed new leprosy patients attending the outpatient department of Dermatology, Venereology, and Leprology in the tertiary care hospital in Uttar Pradesh University of Medical Sciences from July 2022 to January 2024. This study design was an observational study conducted between July 2022 and January 2024.

A total of 231 people (139 males and 92 females) newly diagnosed with leprosy were included. Inclusion criteria consisted of people newly diagnosed with leprosy, irrespective of their sex and age. Exclusion criteria consisted of people with previously diagnosed leprosy, individuals unwilling or uncooperative with the study procedures, and those suffering from terminal illnesses. A detailed history of demographic profile, socioeconomic status, and occupation was taken. In all patients, a comprehensive examination of skin lesions and palpation of peripheral nerves for enlargement was meticulously conducted.

Ethical clearance

The study protocol was reviewed and approved by the Institutional Ethics Committee at Uttar Pradesh University of Medical Sciences (UPUMS), Etawah, India (approval number 107/2023-2024) with specific attention to informed consent, participant safety, and adherence to current treatment protocols. Written informed consent in their native language was obtained from all the participants.

Diagnostic criteria

Diagnosis of leprosy was established through clinical evaluation, histopathological analysis, and bacteriological assessment, adhering to standardized criteria outlined by Ridley and Jopling [4]. World Health Organization (WHO) guidelines were followed for classification into paucibacillary and multibacillary types for treatment purposes.

Data analysis

The data was meticulously recorded, tabulated, and analyzed. Statistical analysis was performed using Microsoft Excel.

## Results

Out of 231 patients, 139 (60.17%) were male and 92 (39.82%) were female. Most cases belonged to the age group 40-59 years 87 (37.66%), followed by 20-39 years 82 (35.49%), >60 years 45 (18.61%), and 0-20 years 19 (8.22%). One hundred and thirty-four patients (58%) were illiterate, and 97(41%99) were literate. Ninety-five (41.12%) patients were from lower socioeconomic strata, 79 (34.19%) were farmers, 51 (22.07%) were laborers, 37 (16.01%) were homemakers, 23 (9.95%) employed, 17 (7.35%) unemployed, 13 (5.62%) students and 11 (4.76%) were businesspeople. One hundred and eighty-eight (81.38%) leprosy patients were residents of urban areas and 198 (85.71%) were married. History of close contact with an afflicted person was present in 34 (14.71%), as shown in Table 1.

**Table 1:** Demographic characteristics of leprosy patients

Demographic characteristics		Frequency	Percentage
Age group	0-20	19	8.22%
	20-39	82	35.49%
	40-59	87	37.66%
	>60	45	18.61%
Gender	Male	139	60.17%
	Female	92	39.82%
Educational	Illiterate	134	58%
	Literate	97	41.99%
Religion	Hindu	203	87.87%
	Muslim	28	12.12%
Place of Residence	Rural	188	81.38%
	Urban	43	18.61%
Marital Status	Married	198	85.71%
	Unmarried	33	14.28%
Contact history	Present	34	14.71%
	Absent	197	85.28%
Socioeconomic Status	Upper	9	3.89%
	Upper middle	17	7.35%
	Lower middle	31	13.41%
	Upper lower	79	34.19%
	Lower	95	41.12%
Occupation	Business	11	4.76%
	Farmer	79	34.19%
	Homemaker	37	16.01%
	Laborer	51	22.07%
	Student	13	5.62%
	Employed	23	9.95%
	Unemployed	17	7.35%

**Figure 1:**
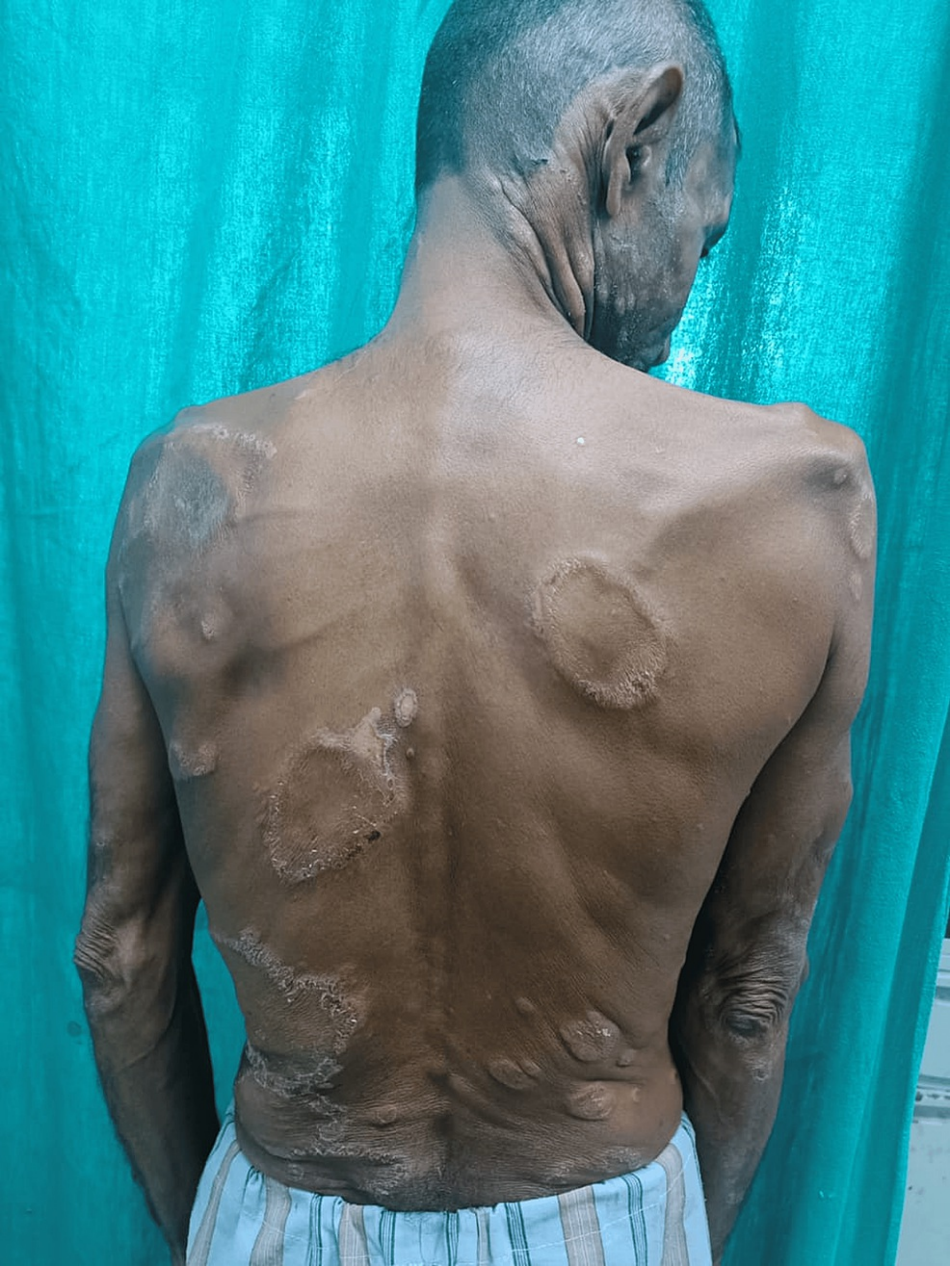
Multiple anhidrotic annular plaques in a patient with lepromatous leprosy

Clinically, most patients belonged to the borderline tuberculoid (BT) type, which was seen in 97 (41.99%) patients, followed by the borderline lepromatous (BL) type, seen in 64 (27.70%) patients. Forty-four (19.04%) patients had lepromatous leprosy (LL) (Figure 1), and 17 (7.35%) had tuberculoid leprosy (TT). Mid-borderline (BB) type was seen in nine (3.89%) patients, as described in Table 2.

**Table 2:** Disease spectrum according to Ridley-Jopling criteria BB, Borderline leprosy; BL, Borderline lepromatous leprosy; BT, Borderline tuberculoid leprosy; LL, Polar lepromatous leprosy; TT, Polar tuberculoid leprosy

Type	Frequency	Percentage
TT	17	7.35%
BT	97	41.99%
BB	9	3.89%
BL	64	27.70%
LL	44	19.04%

According to the WHO classification, 124 (53.67%) patients belonged to the multibacillary type, whereas 107 (46.32%) patients were paucibacillary type (Table 3).

**Table 3:** Classification of leprosy patients according to WHO criteria

Type	Frequency	Percentage
Paucibacillary	107	46.32%
Multibacillary	124	53.67%

Only 24 (10.4%) patients were found positive for *M. leprae* when their slit-skin smear was examined. The remaining 207 (89.6%) were negative for *M. Leprae* (Table 4).

**Table 4:** Bacteriological test results for leprosy patients

Slit skin smear test results		
Result	Frequency	Percentage
Positive	24	10.4%
Negative	207	89.6%

**Figure 2:**
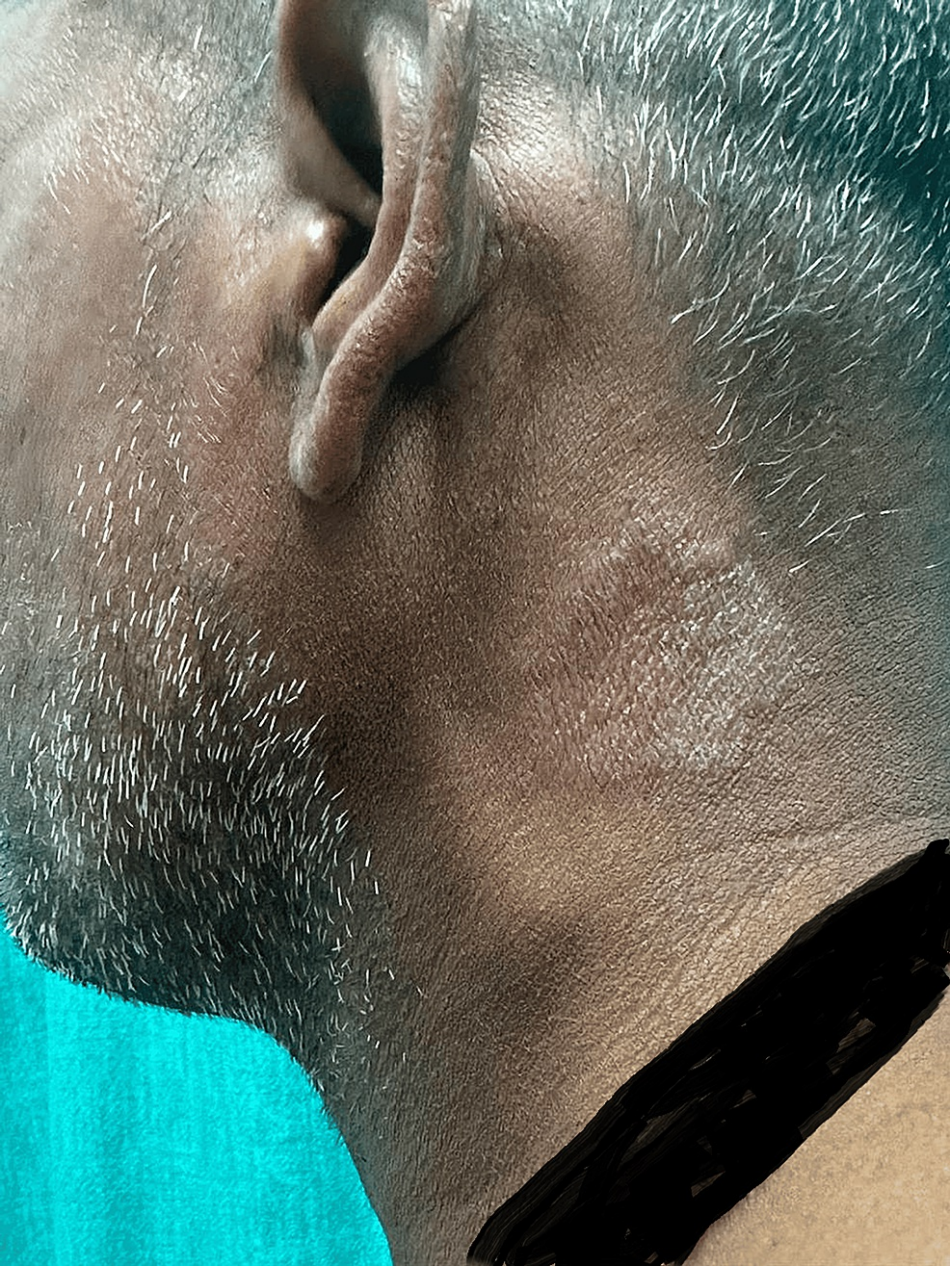
Patient of BT spectrum with left greater auricular nerve thickening BT: Borderline tuberculoid

Multiple nerves were involved in 147 (63.6%) patients. The ulnar nerve was the most common nerve involved, in 63 (27.27%) cases, followed by the common peroneal nerve in 47 (20.34%) cases, the median nerve in 31 (13.41%) cases, the great auricular nerve (Figure 2) in four(1.73%) cases and radial nerve in two (0.86%) cases (Table 5).

**Table 5:** Frequency of nerve involvement, lepra reactions, and deformities in leprosy patients

Various complications in leprosy patients			
Type of complications		Number of patients	Percentage
1. Nerve involvement			
	Ulnar nerve	63	27.27%
	Common Peroneal nerve	47	20.34%
	Median nerve	31	13.41%
	Greater auricular nerve	4	1.73%
	Radial nerve	2	0.86%
2. Lepra reactions			
	Type I	19	8.22%
	Type II	79	34.19%
	No reaction	133	57.57%
3. Deformities			
	Foot drop	13	5.62%
	Claw hand	11	4.76%
	Eye changes	6	2.5%
	Trophic ulcer	34	14.7%
	Palate perforation	1	0.43%

**Figure 3:**
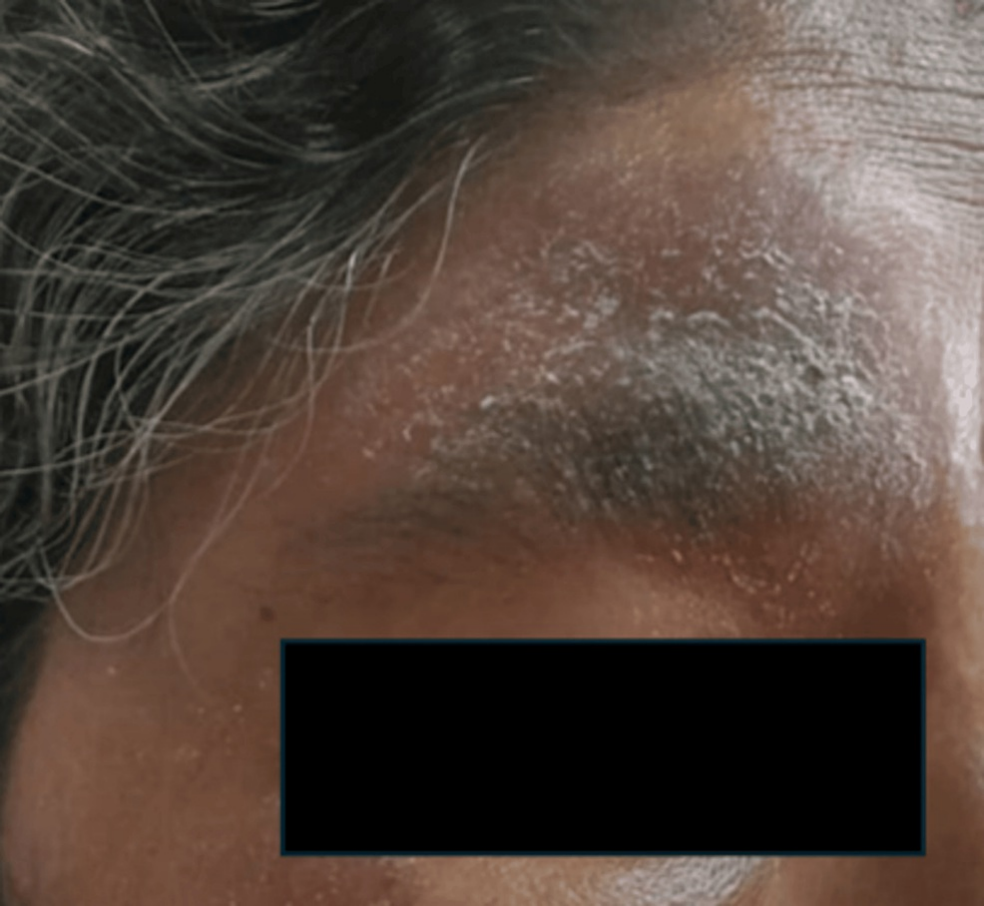
Type 1 Lepra reaction seen at the forehead in a leprosy patient

Type-I lepra reactions (Figure 3) were seen in 19 (8.22%) patients, type-II lepra reactions were seen in 79 (34.19%) patients, and no lepra reactions were seen in the remaining 133 (57.57 %) patients. The majority of the patients showed no lepra reactions (Table 5). 

**Figure 4:**
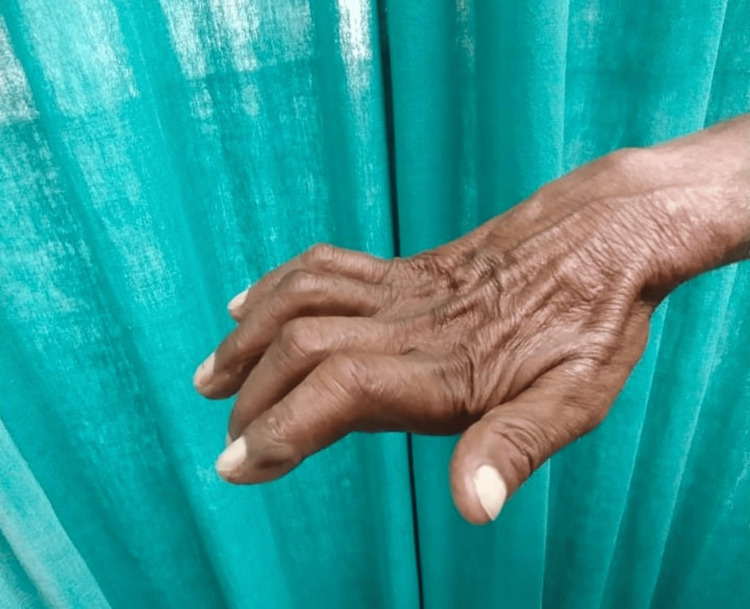
Claw hand deformity due to leprosy

Trophic ulcers were found to be the predominant deformity in 34 (14.7%) patients, followed by foot drop in 13 (5.62 %), claw hand (Figure 4) seen in 11 (4.76%) patients, and eye changes in six (2.5%) patients. Palate perforation was seen in only one (0.43%) patient (Table 5).

**Figure 5:**
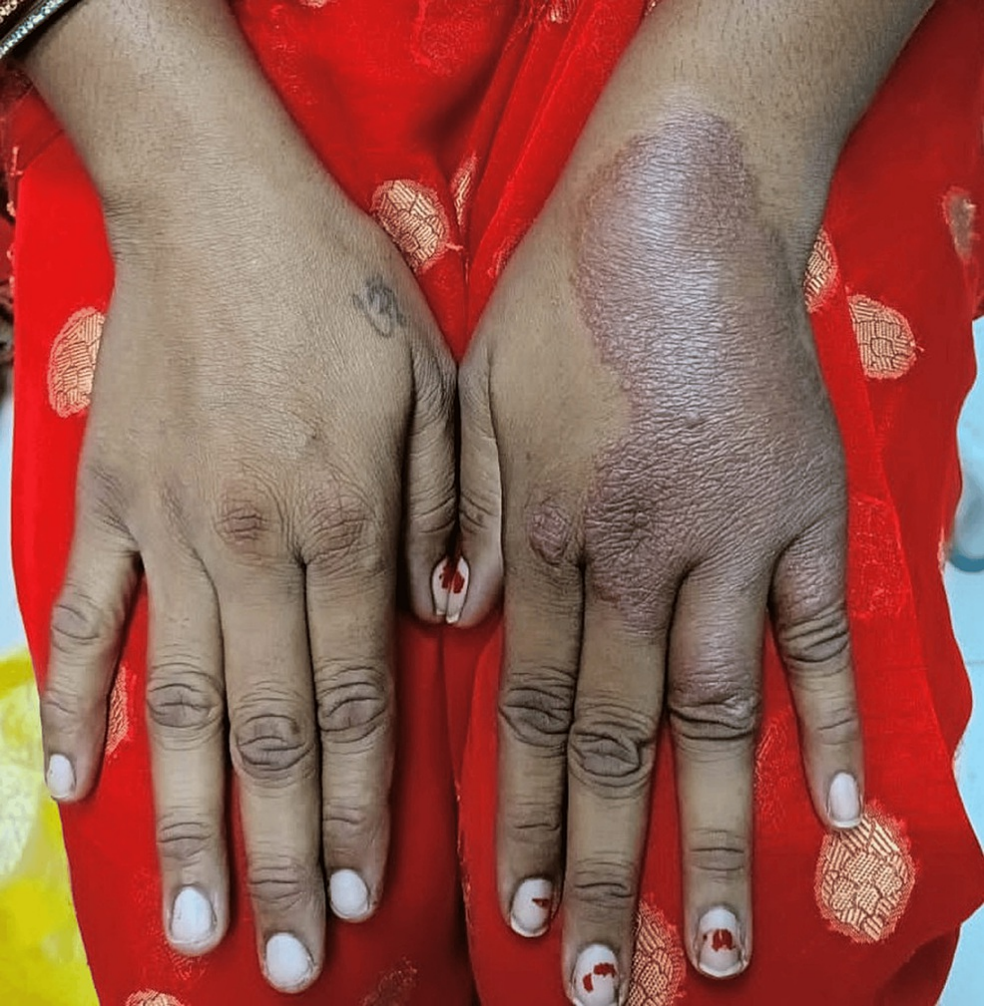
Solitary hypoaesthetic erythematous plaque in a borderline tuberculoid leprosy patient

The most common symptom at presentation was hypoaesthesia 207 (89.6%), followed by skin hypopigmentation (Figure 5) 133 (57.5%) and thickened peripheral nerves 117 (50.64%) (Table 6).

**Table 6:** Clinical presentations in leprosy patients

Sr. No.	Clinical symptoms	Number	Percentage
1.	Epistaxis	7	3.03%
2.	Hypoesthesia	207	89.6%
3.	Trophic ulcer	34	14.71%
4.	Madarosis	21	9.09%
5.	Saddle nose	11	4.76%
6.	Leonine facies	08	3.46%
7.	Ichthyosis	17	8.22%
8.	Eye involvement	6	2.5%
9.	Ear lobe thickening	11	4.76%
10.	Hypopigmented / Erythematous patches	133	57.5%
11.	Thickened peripheral nerve	117	50.64%
12.	Claw hand/foot drop	24	10.38%

## Discussion

India has made significant progress in controlling leprosy, bringing down the number of new cases. However, new cases are still being reported, indicating that challenges remain in eliminating the disease completely. One challenge may be a reduction in resources for leprosy programs after India was declared to have eliminated leprosy. Driven by resource constraints, India integrated its specialized leprosy services into the general healthcare system. This led to a decreased focus on leprosy, potentially affecting early detection and timely treatment. India still accounts for nearly 60% of the world’s incidence of leprosy and it seems that numbers are not going to decline anytime soon [8].

We found that the most affected age group was 40-59 years, a finding similar to the study by Costa et al. [9]. However, in the study by Gupta et al., it was found that the most affected age group was 20-39 years, i.e., the reproductive phase of life in both sexes [10]. Similar observations were also made by other researchers in their studies, like Relhan et al. [11], Kulkarni SK [12], Hazarika et al. [13], and Kumar et al. [14]. This age group being most affected indicates that the population is more vulnerable towards leprosy infection, mostly due to this age group's greater mobility and increased opportunity for the spread of disease by direct contact in a large population. Nevertheless, leprosy in young patients points towards endemicity of the disease [10].

In our study, 139 cases were males and 92 were females. Males outnumbered females, similar to the findings of the study done by Costa et al. [9], Gupta et al. [10], Hazarika et al. [13], Dimri et al. [15], Baraithiya et al. [16], etc. This may be attributed to more exposure and a higher chance of coming into prolonged contact and getting infected as males go for outdoor work more than females. There is also a difference in the treatment-seeking behavior of males and females.

In the present study, most patients were illiterate 134 (58%) and 97 (41%99) were literate. Most were semi-skilled by occupation and belonged to lower socio-economic status. The disease was most common among farmers, 79 (34.19%) followed by laborers which is similar to the study done by Gupta et al. [10], in which they also found that farmers (25.86%) were more commonly affected followed by laborers. This can be associated with factors like low economic status, illiteracy, overcrowding, poor personal hygiene, and malnutrition in agricultural workers and laborers.

Ninety-seven (41.99%) patients belonged to the borderline tuberculoid (BT) type in our study, followed by 64 (27.70%) patients of borderline lepromatous (BL) type. Hazarika et al. [13] reported borderline lepromatous leprosy in 37.9% of patients, followed by lepromatous leprosy in 32.8% of patients. Similar findings were reported in the studies of Shenoi and Siddappa [17] and Singhi et al. [18]. Only 24 (10.4%) of cases in our study were found positive with the slit skin smear test. Most cases were multibacillary 124 (53.67%), and only 107 (36.36%) were paucibacillary type. This finding was also supported by the study of Hazarika et al. [13], who reported that multibacillary cases were the commonest. Multibacillary leprosy (MB) cases are clinically important as they are a major reservoir of infection and are also predisposed to lepra reactions and subsequent deformities [14].

Lepra reactions were noted in 98 (42.4%) patients in the study, with type-II reactions being more than three times more common than type-I reactions, the finding is similar to the studies by Kumar et al. (34.9%) [14], Singal and Sonthalia [19], and Relhan et al. (23.4%) [11]. We also found that type-II reactions were seen in 79 (34.19%) subjects, but no lepra reactions were observed in 133 (57.57%) patients. It is essential to recognize reactional leprosy irrespective of the type of reaction. This is because patients with type I lepra reactions are more prone to deformities, while patients with type II lepra reactions are more prone to systemic complications [10].

In our study, 65 (28.14%) patients had deformities related to leprosy. This finding is similar to the study done by Rathod et al. [20]. Deformities were present in 20.0% of patients in the study by Kumar et al. [14]. Patel et al. [21] reported that 50% of their patients had deformities. Mahajan et al. reported it to be 40.11% [22], Mehta et al. reported 53.33% of patients having deformities [23], and Jindal et al. reported a percentage of deformities to be 54.47% [24].

We found trophic ulcers of the hand or foot in 34 (14.7%) patients, which was the most common morphological deformity. Occurrence of these deformities might be associated with late diagnosis, multibacillary disease due to high bacillary load, improper/ inadequate treatment of reactions/neuritis, and lack of proper counseling [10]. Nerve Involvement is also seen in leprosy patients. The ulnar nerve was the most commonly involved nerve in 63 (27.27%) patients in our study, which is similar to the findings in a study done by Gupta et al. [10] (77.58%) and Hazarika et al. [13] (55%).

Clinical presentation

The most common clinical presentation in the present study was hypoaesthesia in 203 (89.6%), followed by hypopigmented/erythematous patches in 133 cases (57.5%). Hazarika et al. [13] found that the most common skin lesions were plaques followed by macules. Out of the total, 2.5% of leprosy patients presented with involvement of the eye (photophobia, diminished vision, cataract, and conjunctivitis). Ocular features were noted in 13.4% of patients by Kumar et al. [14]. Tegta et al. [25] noted eye involvement in 8.6% of patients with conjunctivitis as a common presentation. Jindal et al. [24] also found anterior uveitis in eight patients.

We observed that in a short span of a few months, many new cases of leprosy were reported and referred to our OPD, with clinical presentations ranging from the involvement of skin, nerves, eyes, and deformities. This unseen burden of disease is causing the failure of the healthcare system to eradicate leprosy completely. There should be continued efforts to educate the masses and healthcare professionals regarding leprosy, its symptoms, and the importance of post-exposure prophylaxis.

Limitations

Our study was limited to a single center, and the duration was 18 months. Only people reporting themselves to the hospital were included. Observing for a longer period might have resulted in much better statistical outcomes. Furthermore, a community-based approach for a longer duration, involving contact tracing and actively seeking new cases would have better epidemiological implications, but this was outside the design of the study.

Although the number of cases of this disease is low overall, many patients suffering from it still turn up in our daily OPD clinic. These patients are sometimes referred from other OPDs, whereas some patients are overlooked in other OPDs and present late to specialized clinics. Hence, this study is an important step towards recognizing the remaining burden of leprosy and making healthcare workers aware of the current epidemiological scenario to help them refer such patients early. This will not only expedite early treatment and lessen the deformities caused but will also impact society by lessening the stigma these people endure.

## Conclusions

Although leprosy has diminished in incidence as a public health concern, it is still present in society and needs to be taken care of by health workers. The present study underscores the multifaceted approach needed for tackling leprosy effectively. By integrating clinical and histopathological examinations, spreading awareness, and enhancing diagnostic capabilities, healthcare professionals can work towards reducing the burden of leprosy, improving the overall health outcomes and stigma associated with affected people in the region under study and the whole population at large.
